# Teplizumab: a promising intervention for delaying type 1 diabetes progression

**DOI:** 10.3389/fendo.2025.1533748

**Published:** 2025-04-28

**Authors:** Muhammad Rizwan Saleem, Muhammad Talha Khan

**Affiliations:** Dow Medical College Bachelor of Medicine, Bachelor of Surgery (MBBS), Dow University of Health Sciences, Karachi, Sindh, Pakistan

**Keywords:** type 1 diabetes, mechanism of diabetes, progression of diabetes, diabetes treatment, teplizumab, immunotherapy, anti-CD3 antibody

## Abstract

Type 1 diabetes (T1D) arises from an autoimmune attack on pancreatic beta cells, leading to a reliance on external insulin to maintain glucose levels. In recent years, research has increasingly focused on preventive strategies for individuals at high risk. A promising new intervention in this field is Teplizumab, the first approved disease-modifying therapy for T1D. Teplizumab is designed to delay progression to stage 3 T1D in adults and children aged 8 years and older who are diagnosed with stage 2 T1D.

## Introduction

Diabetes is the most prevalent noncommunicable chronic illness, currently affecting approximately 420 million people worldwide, with estimates suggesting an increase to 640 million by 2040. Among two main types, type 1 diabetes (T1D) typically presents in childhood or adolescence and accounts for approximately 80% of new diagnoses in individuals aged 19 years or younger. Patients with T1D require lifelong insulin therapy to survive due to the body’s immune system attacks and damage insulin-producing beta cells in the pancreas. This autoimmune response primarily affects genetically predisposed individuals, particularly those carrying the HLA-DQ2 and HLA-DR2 genes or with family members who have T1D (1).

The risk of developing T1D is further influenced by environmental factors, including obesity and certain infectious agents such as measles, mumps, and influenza viruses, which are particularly prevalent in regions like South Asia, where vaccine hesitancy among parents is common (2). The decline in insulin production may be rapid or gradual. Adults with T1D often retain some degree of insulin production, as reflected by measurable C-peptide levels, which can contribute to improved glycemic control. In contrast, younger individuals with T1D are at increased risk for complications such as diabetic ketoacidosis and non-ischemic cardiovascular disease (3).

The autoimmune progression in T1D unfolds in distinct stages. Stage 1 is characterized by the presence of two or more antibodies—typically against islet cell cytoplasm, glutamate decarboxylase, insulinoma antigen 2, or zinc transporter 8—without abnormal glucose levels (4). This advances to Stage 2, which is characterized by dysglycemia with the persistence of two or more autoantibodies, and eventually reaches Stage 3, where hallmark symptoms of diabetes emerge, including diabetic ketoacidosis, polydipsia, polyphagia, polyuria, and weight loss (5).

## How type 1 diabetes occurs: a closer look

Years of research suggest that immune-mediated destruction of pancreatic β-cells involves B cells, CD4+ T cells, and CD8+ T cells (6), with CD8+ T cells considered the likely effectors in this destruction. T cell precursors are formed in the bone marrow and migrate to the thymus, where they undergo central tolerance through negative selection, allowing them to distinguish self from non-self-antigens. Selected T cells then circulate in the bloodstream, eventually reaching lymph nodes where they interact with specific peptides. In type 1 diabetes, β-cell peptides are presented by antigen-presenting cells (APCs) to autoreactive CD4+ T-lymphocytes within the lymph nodes of the pancreas (7).

As T1D develops, immune cells infiltrate the pancreas, targeting insulin-producing β-cells and creating an inflammatory environment called insulitis. This inflammation accelerates disease onset by amplifying islet antigen presentation via HLA class I molecules and the migration of activated T cells to the pancreatic islets, where they initiate β-cell destruction, a process further exacerbated by proinflammatory cytokines released from other immune cells, including monocytes, natural killer cells, neutrophils, and macrophages (8).

Under normal conditions, self-reactive T cells are removed by peripheral tolerance mechanisms, largely mediated by regulatory T cells (Tregs), which prevent tissue damage. However, defects in Treg function can exacerbate β-cell damage in the pancreas. Beyond this localized immune response, T cells can activate humoral immune cells, particularly B cells, leading to the production of autoantibodies against β-cell proteins. These autoantibodies, which are detectable in serum, are essential biomarkers for T1D diagnosis and progression (9).

## Existing therapies: falling short?

Current treatments for T1D primarily rely on lifelong exogenous insulin and certain medications to maintain optimal blood glucose levels. Despite ongoing efforts to enhance insulin regimens, current hormone replacement therapies offer only symptomatic relief, without affecting the underlying disease pathogenesis or long-term outcomes. Recently, the focus has shifted toward preventive approaches for individuals at high risk. Research suggests that immunomodulatory therapy, when administered early in the disease course, may help preserve β-cell function by protecting these cells from autoimmune attack (10).

## How teplizumab works: targeting type 1 diabetes at its source

Various immune interventions, when investigated in patients with newly diagnosed clinical type 1 diabetes, have been shown to slow the reduction in beta-cell function. One such drug is Teplizumab, the first disease-modifying therapy for T1D approved by the Food and Drug Administration (FDA), which is designed to delay the onset of stage 3 T1D in adults and adolescents already at stage 2. Teplizumab, a new humanized anti-CD3 monoclonal antibody that mitigates autoimmune destruction of pancreatic β-cells through increases in proportion of regulatory T cells and exhausts CD8+ T cells and CD4+ T-cell in peripheral blood, leading to deactivation of autoreactive T lymphocytes, which in turn moderates the immune response and slows down the progression (11).

**Figure 1:**
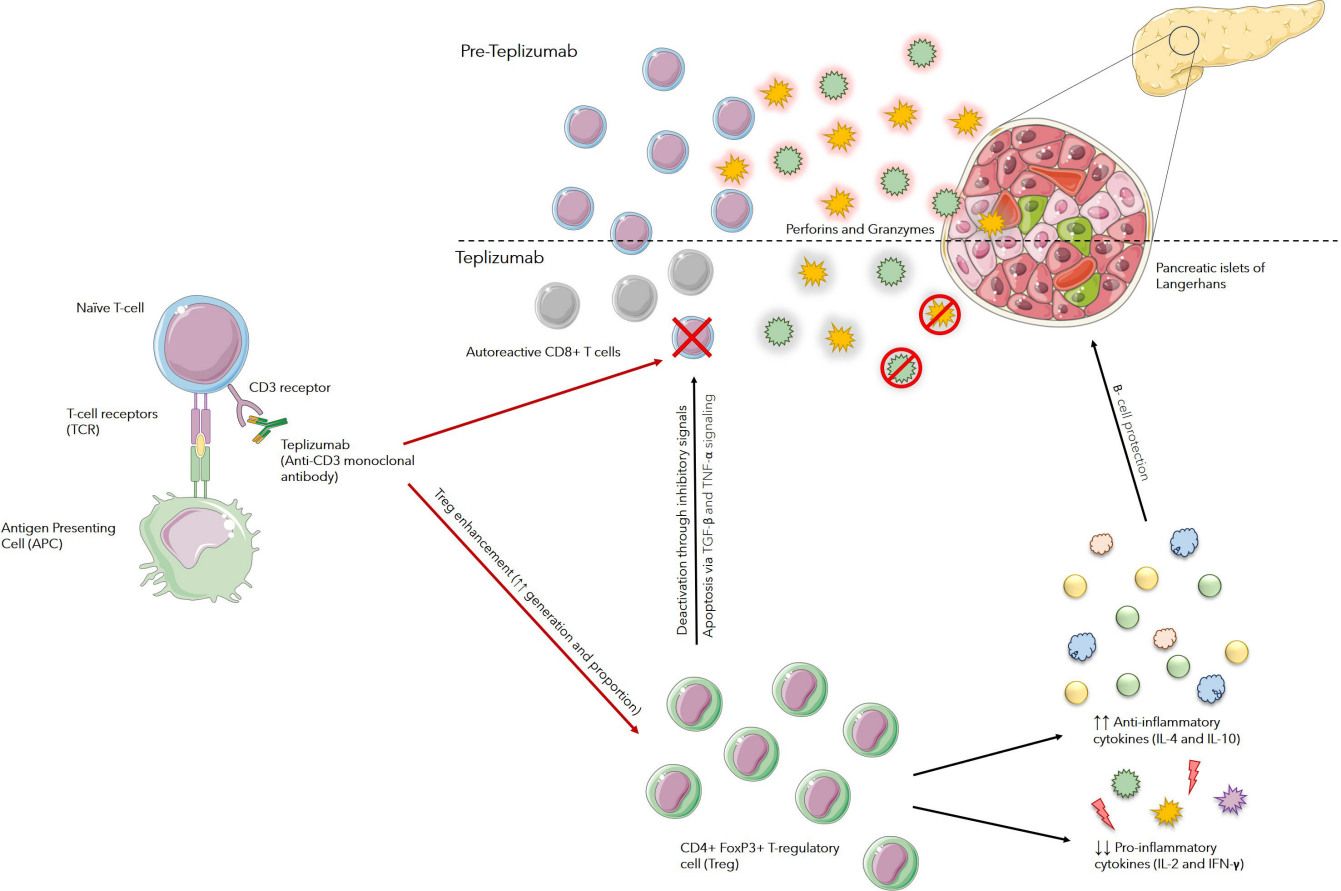
Mechanism of Action of Teplizumab: Teplizumab binds to CD3 receptors on the surface of T cells and modulates T-cell receptor (TCR) signaling. This partial signaling leads to an increase in regulatory T cells (Tregs) and anergy or apoptosis of autoreactive T cells. Enhancement in function and number of Tregs suppress autoreactive immune responses against beta cells, mainly by increasing anti-inflammatory cytokines, such as IL-4 and IL-10, and decreasing pro-inflammatory cytokines such as IL-2 and IFN-γ. Tregs also cause suppression of autoreactive T-cells through various pathways, including apoptosis in activated T cells through signaling via TGF-β and TNF-α pathways. This limits their ability to proliferate and attack beta cells.

These effects are thought to occur through various pathways, including apoptosis in activated T cells through signaling via TGF-β and TNF-α, altering activity of receptor complex found on surface of T-cells, change of T-cells migration patterns and adjustment of T-cell tolerogenic mechanisms. Additionally, teplizumab promotes the generation of CD4+FoxP3+ T cells, which suppress the production of proinflammatory cytokines such as IL-2 and IFN-γ, while simultaneously facilitating the release of anti-inflammatory cytokines like IL-4 and IL-10. These actions suggest that teplizumab may provide significant therapeutic benefits for individuals with T1D (12) (**Figure 1**).

## Testing teplizumab: the road through clinical trials

Over the last twenty years, numerous clinical studies on teplizumab’s effects on individuals with stage 3 of type 1 diabetes have demonstrated the retention of β-cell function, as evaluated through the C-peptide concentration. In phase 2, randomized, placebo-controlled, double-blind trial, teplizumab delayed progression to clinical type 1 diabetes in people who were considered to be at high risk because they had relatives with the disease. The study included 76 participants aged 8 years and older, who were assigned to receive either a single 14-day course of teplizumab or a placebo. Fifty-five participants (72%) were ≤18 years of age. Glucose tolerance tests were performed at 6-month intervals for follow-up to monitor progression to type 1 diabetes.

Over a median follow up period of 51 months, 43% of patients in Teplizumab group progressed to stage 3 of T1D, compared to 72% in the placebo group. The median time to diagnosis varied significantly between the two groups: 48.4 months for those receiving teplizumab and 24.4 months for the placebo group (hazard ratio 0.41, 95% CI 0.22–0.78, P = 0.006). The most pronounced effect of teplizumab was observed within the first year, during which only 7% of the participants in the treatment group were diagnosed with disease, compared to 44% of those in the placebo group (13). In an extension trial with a median follow-up of 76.9 months, the median time to diagnosis was 59.6 months for the teplizumab group and 27.1 months for the placebo group. Among the participants, 50% of those treated with teplizumab remained free of diabetes, in contrast to 22% of those receiving the placebo (14).

Similar results were achieved in another phase 3, randomized, placebo-controlled trail which enrolled 328 children and adolescent with T1D. By week 78, patients in the teplizumab group exhibited significantly higher stimulated C-peptides level compared to those who were given placebo. However, no notable differences were found in other endpoints, such as glycated hemoglobin levels, insulin dosage, or the annual frequency of hypoglycemic events (15).

## Side effects to consider

The most frequently reported adverse effects in more than 10% of participants included lymphopenia (73%), rash (36%), leukopenia (21%), and headache (11%). Precautions for teplizumab include premedication and monitoring for cytokine release syndrome (CRS), which typically occurs within the first five days of treatment. CRS affected 5% of patients receiving teplizumab compared with 0.8% in the placebo group and manifested as fever, nausea, fatigue, headache, myalgia, arthralgia, elevated ALT, AST, and bilirubin levels. Teplizumab should be discontinued if ALT or AST levels exceeds five times the upper limit of normal, and treatment may need to be paused if severe CRS occurs. Additionally, it should not be administered to individuals with an active serious infection, and treatment should be halted if such an infection develops.

## Discussion

The emergence of new therapies such as Teplizumab, capable of delaying or preventing type 1 diabetes, presents a significant opportunity to reshape the trajectory of this condition. A survey conducted to assess parents’ views on Teplizumab, a therapy designed to delay the onset of Type 1 Diabetes (T1D) involved 95 parents of children with T1D who also had at least one other child without the condition. Findings revealed that only 6.3% of participants had a detailed understanding of Teplizumab, yet 52.6% were willing to approve its use if their child met the eligibility criteria. When asked about benefits of using Teplizumab for delaying disease progression, 62.1% reported reduced stress for families, 40% noted prevention of DKA, and increased readiness to manage diabetes was reported by 27.4% of parents. Emotional responses were mixed, with 53.7% of parents expressing hope about the treatment, 52.6% were concerned about potential negative emotional impacts during its administration (16).

While transient adverse events, such as cytokine release syndrome and lymphopenia, rash have been reported, the overall safety profile appears favorable, particularly when compared with conventional immunosuppressive therapies used earlier for treatment of T1D such as azathioprine, cyclosporine and prednisone which reduce the insulin requirements and overall state of health for brief period of time but have serious hazards with long term use that voiced serious objection (14). Currently, the clinical use of teplizumab is limited to relatives of individuals with T1D with dysglycemia who have two or more islet autoantibodies yet have not progressed to overt diabetes. Further research involving an extensive and diverse population, particularly the pediatric age group, as its effect has yet to be observed in individuals younger than 8 years of age is imperative to fully understand its long-term benefits and risks. These studies will also help to evaluate the optimal dosing regimen, treatment duration, and timing of teplizumab administration to maximize therapeutic efficacy while minimizing potential adverse effects. Even though these challenges and uncertainties remain, the remarkable progress made to date gives us hope for a future where T1D will no longer be associated with lifelong dependence on insulin therapy.

Besides focusing on improving the medical treatment for T1D, special emphasis must be given to screening programs with the aim to detect the presymptomatic stages of the disease by identifying specific islet autoantibodies in individuals at risk. This early identification offers numerous advantages, primarily reducing the risk of diabetic ketoacidosis (DKA) at diagnosis. Studies show that DKA rates drop to less than 5% in screened populations compared to 15-80% in unscreened individuals. This early detection also provides families and healthcare providers more time to prepare for disease management, offers emotional relief, opens the doors to therapeutic interventions, preserves beta cell function and improves long term outcomes (17).

## Data Availability

The original contributions presented in the study are included in the article/supplementary material. Further inquiries can be directed to the corresponding author.
